# The Histone Modification Code in the Pathogenesis of Autoimmune Diseases

**DOI:** 10.1155/2017/2608605

**Published:** 2017-01-03

**Authors:** Yasuto Araki, Toshihide Mimura

**Affiliations:** ^1^Department of Rheumatology and Applied Immunology, Faculty of Medicine, Saitama Medical University, Saitama, Japan; ^2^Project Research Division, Research Center for Genomic Medicine, Saitama Medical University, Saitama, Japan

## Abstract

Autoimmune diseases are chronic inflammatory disorders caused by a loss of self-tolerance, which is characterized by the appearance of autoantibodies and/or autoreactive lymphocytes and the impaired suppressive function of regulatory T cells. The pathogenesis of autoimmune diseases is extremely complex and remains largely unknown. Recent advances indicate that environmental factors trigger autoimmune diseases in genetically predisposed individuals. In addition, accumulating results have indicated a potential role of epigenetic mechanisms, such as histone modifications, in the development of autoimmune diseases. Histone modifications regulate the chromatin states and gene transcription without any change in the DNA sequence, possibly resulting in phenotype alteration in several different cell types. In this paper, we discuss the significant roles of histone modifications involved in the pathogenesis of autoimmune diseases, including rheumatoid arthritis, systemic lupus erythematosus, systemic sclerosis, primary biliary cirrhosis, and type 1 diabetes.

## 1. Introduction

A loss of self-tolerance causes autoimmunity in which the aberrant immune system attacks the healthy cells and tissues, leading to chronic inflammation. The immune system requires a strict balance of stable and reversible gene expression to maintain the normal function of immune cells and to ward off the development of autoimmune diseases. A gain of autoreactivity in immune cells as well as a loss of suppressive functions in regulatory T cells (Tregs) has been suggested to be implicated in the autoimmune pathogenesis. Recently, it has been demonstrated that not only genetic and environmental factors but also epigenetic changes are involved in the etiology of autoimmune diseases. Epigenetic mechanisms, such as histone modifications, DNA methylation, and microRNAs (miRNAs) signaling, contribute to the maintenance of the normal immune response through the dynamic regulation of chromatin structure as well as gene transcription. Epigenetic dysregulation may modulate the functions of immune cells, resulting in autoimmunity. Therefore, epigenetic regulation is at present focused on in the field of autoimmune diseases. However, a number of different histone modifications exist and their interactions are complex. Thus, the studies of histone modifications in autoimmune diseases are limited, compared with DNA methylation and miRNAs that have been extensively investigated. Histone modifications have a potential for biomarkers and therapeutic targets of autoimmune diseases. This review summarizes the recent advances in the studies of the roles of histone modifications in autoimmune diseases, including rheumatoid arthritis (RA), systemic lupus erythematosus (SLE), systemic sclerosis (SSc), primary biliary cirrhosis (PBC), and type 1 diabetes (T1D).

## 2. The Pathogenesis of Autoimmune Diseases

Autoimmune diseases are multifactorial disorders characterized by the loss of immunological tolerance to self-antigens and the presence of autoantibodies and/or autoreactive T and B cells. The autoimmune inflammation can involve multiple organs, resulting in systemic autoimmune diseases, such as RA, SLE, and SSc. On the other hand, organ-specific autoimmune diseases, including PBC and T1D, occur when the autoimmune responses are limited to specific organs. To date, more than 80 specific autoimmune diseases have been identified. In 1957, Witebsky et al. defined an autoantibody based on certain criteria, such as (1) the direct demonstration of circulating antibodies that are active at body temperature or of cell-bound antibodies by indirect means, (2) the recognition of the specific antigen against which this antibody is directed, (3) the production of antibodies against the same antigen in experimental animals, and (4) the appearance of pathological changes in the corresponding tissues of an actively sensitized experimental animal that are basically similar to those in human disease [1]. In 1963, Mackay and Burnet defined autoimmune diseases in their* Autoimmune Diseases* textbook as “a condition in which structural or functional damage is produced by the action of immunologically competent cells or antibodies against normal components of the body” that was induced by the emergence of “forbidden” (autoreactive) clones of lymphocytes [2]. In addition, they noted that the diseases were characterized by (1) autoantibodies, (2) hypergammaglobulinemia, (3) tissue deposition of immune complexes, (4) lymphocytic and plasma cell accumulation in the affected tissues, (5) the therapeutic benefit from corticosteroids, and (6) the overlap of differing autoimmune manifestations in the same patient. Previously, Burnet had proposed the clonal selection theory, in which antigen “C” selects “C”-specific lymphocytes and stimulates their proliferation, as either antibody-producing plasma cells or memory cells, and was awarded the Nobel Prize for discovery of acquired immunological tolerance in 1960 [3]. Based on this theory, immunological self-tolerance is caused by the deletion of self-reactive clones, whereas autoimmunity arises by the emergence of self-reactive clones [4]. In 1995, Sakaguchi et al. identified CD4^+^CD25^high^ Tregs that suppress the functions of CD4^+^ effector T cells [5]. At present, Tregs, which were later shown to be FOXP3^+^ cells, are thought to maintain immunological self-tolerance and prevent autoimmune diseases [6].

**Figure 1:**
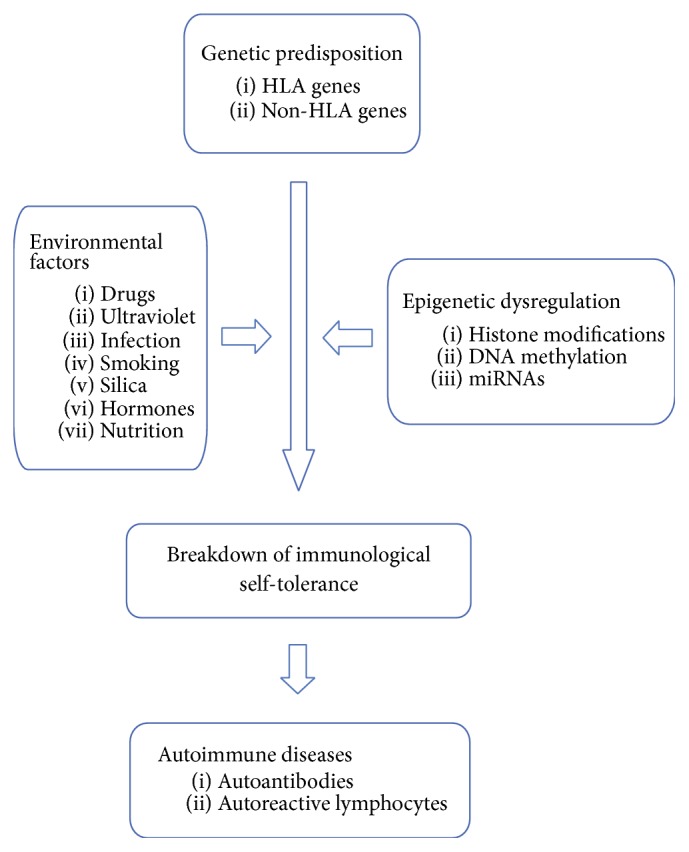
In genetically predisposed individuals, several environmental factors, along with aberrant epigenetic mechanisms, induce a loss of immunological self-tolerance, resulting in the development of autoimmune diseases.

It is postulated that environmental elements trigger autoimmune diseases in genetically predisposed individuals [7]. A number of genome-wide association studies have demonstrated that the susceptibility to autoimmune diseases is affected by multiple risk genes, including human leukocyte antigen (HLA) genes as well as non-HLA genes that are related to cellular and humoral immune responses [8–13]. Several studies have shown high concordance rates in monozygotic twins compared with dizygotic twins or sibling pairs, indicating a strong contribution of a genetic component in autoimmune diseases [14]. However, the disease concordance in monozygotic twins is incomplete, suggesting the presence of other factors, such as environmental and epigenetic ones [15, 16] (Figure 1). In fact, environmental factors, such as drugs, ultraviolet exposure, infection, cigarette smoking, crystalline silica, reproductive hormones, and nutrition, have been shown to contribute to the induction of autoimmune diseases through epigenetic changes [17, 18]. The epigenetic mechanisms link the genetic and environmental factors responsible for the onset and development of autoimmune diseases [19–22]. Epigenetic changes, such as histone modifications, in immune cells may cause a breakdown of immunological self-tolerance and lead to the perpetuation or exacerbation of autoimmune diseases [23–26].

## 3. Histone Modifications

### 3.1. Epigenetics and Chromatin Structure

In 1942, Conrad H. Waddington in his* Principles of Embryology* textbook coined the term “epigenetics” to designate a process in which gene regulation modulated development. In 2008, the definition of epigenetics was revised in the Epigenetic Meeting held by the Banbury Conference Center and Cold Spring Harbor Laboratory to “a stably heritable phenotype resulting from changes in a chromosome without alterations in the DNA sequence” [27]. The heritability of epigenetics means the transmission of an epigenetic state through either mitosis or meiosis. In the meeting, three categories of signals were proposed to be involved in the establishment of a stably heritable epigenetic phenotype. The “Epigenator” is an extracellular signal from the environment that triggers an intracellular epigenetic pathway. The “Epigenetic Initiator,” such as a DNA-binding protein and a noncoding RNA, is activated by the Epigenator and determines the precise chromatin location for the establishment of the epigenetic pathway. The “Epigenetic Maintainer,” including histone modifications, DNA methylation, histone variants, and nucleosome positioning, sustains the chromatin state in the initial and subsequent generations.

Genomic DNA is tightly packaged in chromatin by both histone and nonhistone proteins in the nucleus of eukaryotic cells [28]. The basic chromatin subunits are nucleosomes that are comprised of two copies each of the core histone proteins H2A, H2B, H3, and H4, around which 147 base pairs of DNA are wrapped 1.6 times [29, 30]. Histone proteins are comprised of a structured globular domain and a flexible and charged NH_2_-terminus, termed the histone tail, which protrudes from the nucleosome [31]. The chromatin structure can be divided into two distinct categories based on the perspective of the association with gene transcription [32]. “Euchromatin” is an open chromatin structure that affords accessibility for transcription factors to DNA, resulting in gene activation. In contrast, “heterochromatin” is a closed chromatin structure with a low interaction between transcription factors and the genome, leading to gene repression. Epigenetic mechanisms alter chromatin structure and consequently modulate gene transcription in the absence of any change in the DNA sequence. The chromatin structure in the regulatory regions of genomic DNA, such as promoters, enhancers, and silencers, controls gene transcription by modulating the accessibility for transcription factors.

### 3.2. Histone Modifications and Histone Code Hypothesis

In 2000, Strahl and Allis proposed the histone code hypothesis, which says that “multiple histone modifications, acting in a combinatorial or sequential fashion on one or multiple histone tails, specify unique downstream functions” [33]. Distinct patterns of covalent posttranslational modifications in histone tails suggest that a histone “language” may be encoded in these histone modifications, which are read by chromatin-associated proteins and translated into biological functions. They refer to this language as the “histone code.” Histone modifications have been shown to control dynamic transitions between transcriptionally active or silent chromatin states and regulate the transcription of genetic information encoded in DNA (the “genetic code”) [34]. The histone code is suggested to extend the genetic code. Recently, a genome-wide analysis proved that combinatorial patterns of histone acetylation and methylation cooperatively regulate the chromatin state in humans [35].

**Figure 2:**
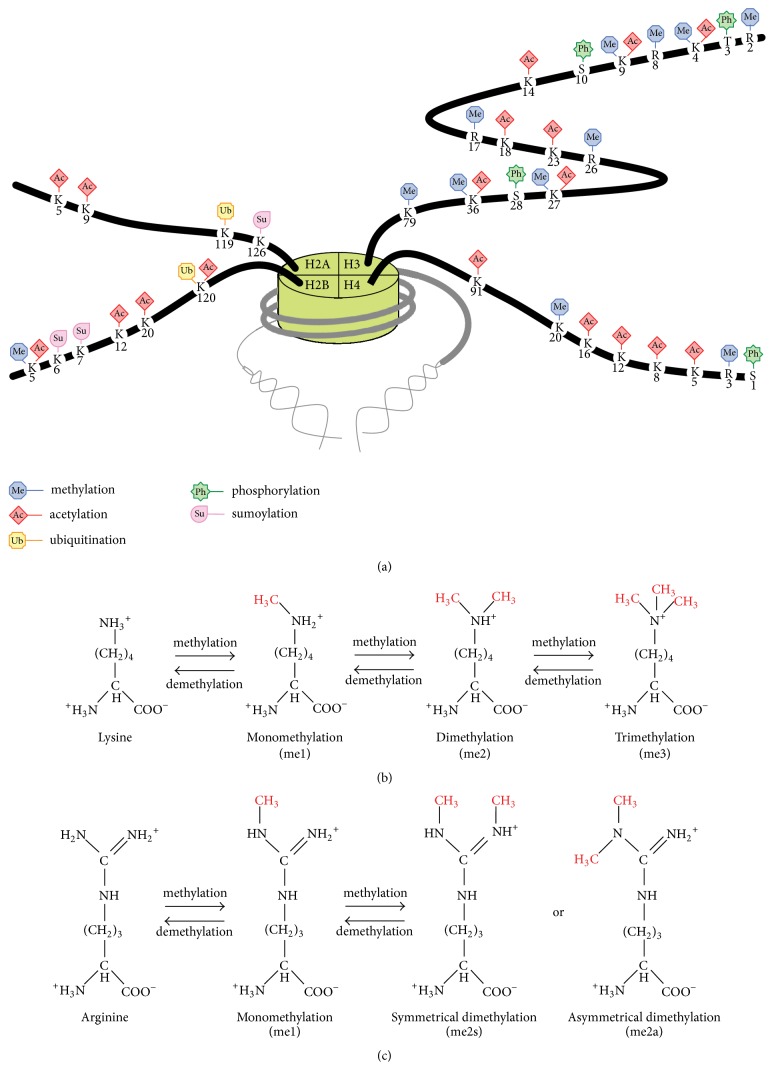
(a) Histone modifications, including methylation, acetylation, ubiquitination, phosphorylation, and sumoylation, have various biological functions, such as the regulation of chromatin states and gene transcription. (b) Lysine residues of histone tails are subject to monomethylation (me1), dimethylation (me2), or trimethylation (me3). (c) Arginine residues of histone tails are subject to monomethylation (me1), symmetrical dimethylation (me2s), or asymmetrical dimethylation (me2a).

Histone modifications, which include methylation, acetylation, ubiquitination, phosphorylation, and sumoylation, are classified as transcriptionally active or repressive markers [36–41] (Figure 2(a)). Analyses of genome-wide profiles of histone modifications and gene expression demonstrated four distinct types of correlations (repressed, active, poised, and bivalent) [42, 43]. In the repressed state, gene transcription is suppressed in a closed chromatin configuration. In the active state, gene transcription is active in an open chromatin configuration. In the poised state, the chromatin is open, but gene transcription is nevertheless low at rest [44]. However, following activation, gene transcription increases rapidly. Chromatin in the bivalent state contains high levels of both active and repressive histone markers and is able to change to an open or closed state both through cell differentiation and upon activation.

### 3.3. Histone Methylation (Table 1)

Histone methylation occurs at specific lysine or arginine residues on histone tails [45, 46]. Histones H2B lysine 5 (H2BK5), H3K4, H3K9, H3K27, H3K36, H3K79, and H4K20 are subject to monomethylation (me1), dimethylation (me2), or trimethylation (me3) on their *ε*-amino groups of lysine residues (Figure 2(b)). Histones H3 arginine 2 (H3R2), H3R8, H3R17, H3R26, and H4R3 undergo monomethylation (me1), symmetrical dimethylation (me2s), or asymmetrical dimethylation (me2a) on their guanidinyl groups of arginine residues (Figure 2(c)). Histone methylation is associated with either transcriptional activation or repression [47]. The functional effects of histone methylation are affected by both the position of the modified residues and the number of methyl groups [48].

Histone methyltransferases (HMTs) transfer methyl groups from S-adenosylmethionine (also called AdoMet or SAM) to either lysine or arginine residues, whereas histone demethylases (HDMs) remove methyl groups [49, 50]. The HMTs and HDMs specifically catalyze particular lysine or arginine residues. The HMTs that catalyze lysine residues are grouped into the (Su(var)3-9, Enhancer of Zeste, Trithorax) SET domain-containing enzyme families (KMT1-3 and KMT5-7), the KMT4/DOT1 family, and others. The HDMs that catalyze lysine residues include the flavin adenine dinucleotide- (FAD-) dependent monoamine oxidase family (KDM1/LSD), the Jumonji C domain-containing demethylase families (KDM2-6), and others. The HMTs that catalyze arginine residues are protein arginine methyltransferases (PRMTs), which are categorized into types I, II, and III in mammalian cells. Type I PRMTs (PRMTs 1, 2, 3, 4, 6, and 8) catalyze the formation of monomethylarginine and asymmetric dimethylarginines. Type II PRMTs (PRMTs 5 and 7) catalyze the formation of monomethylarginine and symmetric dimethylarginines. PRMT7 also belongs to the type III PRMTs that solely catalyze monomethylarginine. HDMs that catalyze arginine residues have not been reported.

#### 3.3.1. H2BK5 Methylation

H2BK5 monomethylation is associated with active promoters, suggesting that H2BK5me1 is an active histone marker [48]. HMTs and HDMs that catalyze H2BK5 have not been reported.

#### 3.3.2. H3K4 Methylation

All of the three states of H3K4 methylation (H3K4me1, H3K4me2, and H3K4me3) surrounding the transcription start sites (TSSs) are reportedly elevated and positively correlated with gene expression [42, 48]. The level of H3K4me3 is elevated in highly active genes, while the levels of H3K4me1 and H3K4me2 are high in intermediately active genes. H3K4 is methylated by the KMT2 family (MLL1, MLL2, MLL3, MLL4, MLL5, SET1A, SET1B, and ASH1L) and the KMT7 family (SET7/9) as well as SMYD3, PRDM9, and PRMT6. H3K4 is demethylated by the KDM1 family (LSD1 and AOF1), the KDM2 family (FBXL10), and the KDM5 family (JARID1A, JARID1B, JARID1C, and JARID1D) as well as JARID2 and NO66.

#### 3.3.3. H3K9 Methylation

H3K9 methylation is considered to play a critical role in the formation of transcriptionally silent heterochromatin and the stable inheritance of the heterochromatin state [51]. Unexpectedly, high levels of H3K9me1 were detected in active promoters, suggesting that this modification is associated with transcriptional activation, even though the levels of both H3K9me2 and H3K9me3 were shown to be increased in silenced genes [48]. H3K9 is methylated by the KMT1 family (SUV39H1, SUV39H2, G9A, GLP, SETDB1, and SETDB2) as well as PRDM1, PRDM2, and PRDM4. H3K9 is demethylated by the KDM1 family (LSD1), the KDM3 family (JMJD1A and JMJD1B), and the KDM4 family (JMJD2A, JMJD2B, JMJD2C, and JMJD2D) as well as JMJD1C, PHF8, and JHDM1D.

#### 3.3.4. H3K27 Methylation

It is suggested that the methylation of H3K27 is associated with gene repression [42]. According to a genome-wide analysis, the levels of H3K27me2 and H3K27me3 are elevated in silent promoters and reduced in both active promoters and genic regions, whereas the level of H3K27me1 is high in active promoters [48]. H3K27 is methylated by the KMT6 family (EZH1 and EZH2). H3K27 is demethylated by the KDM6 family (UTX and JMJD3), as well as UTY and JHDM1D.

#### 3.3.5. H3K36 Methylation

Because the level of H3K36me3 is high at the promoter site in active genes, H3K36me3 is involved in active transcription [48]. In contrast, the H3K36me1 signal has a low association with active promoters. H3K36 is methylated by the KMT3 family (SETD2 and NSD1) as well as NSD2, NSD3, SMYD1, SMYD2, SMYD3, SMYD4, and SMYD5. H3K36 is demethylated by the KDM2 family (FBXL10 and FBXL11), the KDM4 family (JMJD2A, JMJD2B, and JMJD2C), and NO66.

#### 3.3.6. H3K79 Methylation

H3K79me3 is associated with active transcription in yeast, whereas it is localized at both active and silent promoters in humans [48]. H3K79me1 and H3K79me2 do not have any association with either active or silent promoters. H3K79 is methylated by the KMT4 family (DOT1) and demethylated by PHF8.

#### 3.3.7. H4K20 Methylation

H4K20 methylation is suggested to be associated with repressive chromatin. A recent genome-wide analysis demonstrated that H4K20me3 was associated with heterochromatin [48]. On the other hand, H4K20me1 was shown to be located in the promoters or coding regions of active genes and to colocalize with H3K9me1, suggesting that H4K20me1 is an active histone marker. H4K20 is methylated by the KMT5 family (PR-Set7, SUV4-20H1, and SUV4-20H2) and the KMT7 family (SET7/9). HDMs that catalyze H4K20 have not been reported.

#### 3.3.8. H3R2 Methylation

H3R2me2a is mainly catalyzed by PRMT6 and countercorrelates with the methylation of H3K4, suggesting that H3R2me2a is a repressive marker [52, 53]. However, PRMT6 methylates H3K4 and both H3R2me2a and H3K4me3 markers are likely to coexist [54]. Furthermore, genome-wide analyses have indicated that both H3R2me1 and H3R2me2a are associated with active genes [48, 55]. Thus, the data on the H3R2me2a marker are contradictory, and further studies are required to resolve this issue.

#### 3.3.9. H3R8 Methylation

The H3R8 site is symmetrically methylated by PRMT5. H3R8me2s is related to gene silencing [56, 57]. H3R8me2s is strongly associated with H4R3me2s, because both modifications are catalyzed by PRMT5. The acetylation of H3K9 and H3K14 prevents H3R8 methylation.

#### 3.3.10. H3R17 Methylation

CARM1 asymmetrically methylates H3R17. The level of H3R17me2a is elevated at the promoters of active genes, indicating that this modification is an active histone marker [58, 59].

#### 3.3.11. H3R26 Methylation

Asymmetric H3R26 dimethylation is catalyzed by CARM1 and possibly antagonizes K3K27 methylation, suggesting that H3R26me2a is an active histone marker [46, 58].

#### 3.3.12. H4R3 Methylation

The H4R3me2a marker is generated by PRMT1, PRMT6, and PRMT8 and is associated with active promoters [53, 54, 60]. In addition, H4R3me2a facilitates the subsequent acetylation of the histones H3 and H4 [60–62]. On the other hand, H4R3me2s is catalyzed by PRMT5 and PRMT7 and is located in repressed promoters [56, 57, 63]. Furthermore, H4R3me2s is required for DNMT3A-mediated DNA methylation [64]. Since the first five residues (SGRGK) of the histones H4 and H2A are the same, the functions of H4R3 methylation and H2AR3 methylation are thought to be identical [46].

### 3.4. Histone Acetylation

A line of evidence has established that histone acetylation is basically associated with gene activation [65, 66]. A genome-wide study demonstrated that all forms of histone acetylation (H2AK5ac, H2AK9ac, H2BK5ac, H2BK12ac, H2BK20ac, H2BK120ac, H3K4ac, H3K9ac, H3K14ac, H3K18ac, H3K23ac, H3K27ac, H3K36ac, H4K5ac, H4K8ac, H4K12ac, H4K16ac, and H4K91ac) are positively correlated with gene expression [35]. Although histone acetylation is generally elevated in the promoters of active genes, H3K27ac was shown to be associated with active but not inactive enhancers [67]. Histones contain amino acids with basic side chains that are positively charged and are attracted to genomic DNA that are negatively charged [68]. Histone acetylation eliminates the positive histone charge and decreases the interaction between nucleosomes and DNA. This probably causes the change in chromatin structure from heterochromatin to euchromatin. Histone acetylation involves both the initiation and elongation of gene transcription [69]. Histone acetylation also stabilizes the binding of chromatin remodeling factors at promoter regions and induces the unfolding of nucleosomes as well as reduced nucleosome occupancy [70, 71].

The enzymes that acetylate and deacetylate histones have been identified and suggest that histone acetylation is a rapid and reversible process [72]. The histone acetyltransferases (HATs) transfer acetyl groups from acetyl-coenzyme A (CoA) to the *ε*-amino groups of lysine residues in histone tails, resulting in gene activation [73]. HATs contain a bromodomain that recognizes and binds to histone acetylation, and they are categorized into three major families, GNAT (GCN5 and PCAF), MYST (Tip60 and MOF), and CBP/p300. The histone deacetylases (HDACs) remove acetyl groups from lysine residues, leading to gene silencing. The HDACs are grouped into four classes: class I (HDACs 1, 2, 3, and 8), class II (HDACs 4, 5, 6, 7, 9, and 10), class III (SIRT1, SIRT2, SIRT3, SIRT4, SIRT5, SIRT6, and SIRT7), and class IV (HDAC11) [74, 75]. Class I HDACs have sequence homology to class II HDACs and class IV HDACs but not class III HDACs. Class I, II, and IV HDACs are zinc-dependent, whereas class III HDACs are nicotinamide adenine dinucleotide (NAD)^+^-dependent. Genome-wide mapping of the binding of HATs and HDACs to the human genome demonstrate that these enzymes regulate the activation and repression of transcription, respectively [76].

### 3.5. Histone Ubiquitination

Histone ubiquitination is a process of adding ubiquitin peptides to lysine residues [77]. In eukaryotic cells, the histones H2A and H2B are subject to monoubiquitination [78]. H2AK119 monoubiquitination (H2AK119ub1) is associated with transcriptional repression. On the other hand, H2BK120 monoubiquitination (H2BK120ub1) is enriched in the gene body of transcriptionally active genes, enhances a transcriptional elongation, and induces H3K4me2 and H3K4me3 [79]. The H2A-specific histone ubiquitin ligases are RING1A/RIG1B/BMI1, 2A-HUB, BRCA1/BARD1, and UbcH5c. The H2B-specific histone ubiquitin ligases are RNF20/40, RAD6A/B, and UbcH6. The H2A-specific deubiquitinating enzymes (DUBs) are USP16, USP21, 2A-DUB, and BAP1. The DUBs that catalyze both H2A and H2B are USP3 and USP22.

### 3.6. Histone Phosphorylation

The phosphorylation of H3 threonine 3 (H3T3), H3 serine 10 (H3S10), H3S28, and H4S1 is related to gene activation [37, 80]. The serine/threonine kinases that catalyze H3T3, H3S10, H3S28, and H4S1 are Haspin, MSK1/MSK2/RSK2, MSK1/MSK2, and CKII, respectively.

### 3.7. Histone Sumoylation

Histone sumoylation of H2AK126, H2BK6, and H2BK7 has been shown to antagonize other positive modifications such as histone acetylation, thereby resulting in transcriptional repression [81, 82]. The enzymes that catalyze histone sumoylation have not been reported.

## 4. Histone Modification Disorders in Autoimmune Diseases

### 4.1. RA

Most of the studies of histone modifications in RA have focused on abnormalities in synovial fibroblasts (SFs) or tissues. EZH2, an HMT that catalyze H3K27, was shown to be highly expressed in RASFs and induced by tumor necrosis factor *α* (TNF*α*) via the nuclear factor-kappa B (NF-*κ*B) and Jun kinase pathways [83]. EZH2 targets the secreted fizzled-related protein 1 (SFRP1) gene, an inhibitor of Wnt signaling, and is involved in the activation of RASFs. H3K4me3 is elevated and H3K27me3 is reduced in the SFRP1 promoter. Matrix metalloproteinases (MMPs) degrade articular cartilage and play an important role in joint destruction in RA. The expression of MMP-1, MMP-3, MMP-9, and MMP-13 is high in RASFs and the levels of the active histone marker H3K4me3 are increased, whereas those of the repressive histone marker H3K27me3 are decreased in the MMP promoters in RASFs [84]. Because WD (tryptophan-aspartate) repeat domain 5 (WDR5) is a core subunit of the complex proteins associated with SET1 (COMPASS) or COMPASS-like complexes that catalyze H3K4 methylation, WDR5 is required for the generation of H3K4me3. WDR5 knockdown reduces H3K4me3 as well as expression of the MMPs in RASFs. Interleukin- (IL-) 6 and soluble IL-6 receptor *α* (sIL-6R*α*) enhance expression of MMP-1, MMP-3, and MMP-13 but not MMP-9. Signal transducer and activator of transcription 3 (STAT3), an IL-6-induced transcription factor, were shown to be associated with the MMP-1, MMP-3, and MMP-13 promoters but not the MMP-9 promoter. T-box transcription factor 5 (TBX5) expression is high in RASFs and both H3K4me3 and histone acetylation are increased in the TBX5 promoter in RASFs [85]. High IL-6 expression is associated with high levels of H3ac in the IL-6 promoter in RASFs [86].

Huber et al. reported that nuclear HDAC activity is significantly low in RA synovial tissues, while nuclear HAT activity is not altered in RA synovial tissues [87]. The ratio of HDAC activity to HAT activity is significantly low in RA synovial tissues. The expression of HDAC1 and HDAC2 is reduced in RA synovial tissues. These results suggest that histone hyperacetylation occurs in RA. Kawabata et al. showed that nuclear HDAC activity and HDAC1 expression are significantly increased in RA synovial tissues [88]. Gillespie et al. demonstrated that HDAC activity is significantly increased in peripheral blood mononuclear cells (PBMCs) of RA patients [89]. Both trichostatin A (TSA), a pan-HDAC inhibitor, and MI192, a HDAC3-selective inhibitor, suppress TNF*α* and IL-6 production in RA patients PBMCs. Toussirot et al. reported that both HAT and HDAC activities are not altered in PBMCs of RA patients [90]. Horiuchi et al. showed that HDAC1 is highly expressed in RASFs [91]. Knockdown of HDAC1 results in decreased cell proliferation, increased apoptosis, and an upregulation of TNF*α*-induced MMP-1 production in RASFs. Thus, the results of the investigations of the histone acetylation-modifying enzymes seem to be in disagreement and further studies are needed. Several studies have reported the effect of inhibitors of HDACs and HATs in RA. Interestingly, sirtinol, an HDAC inhibitor, significantly decreased HAT activity in RA patients PBMCs [90]. Certain HDAC inhibitors, including TSA, sodium phenylbutyrate, and nicotinamide, have been shown to decrease IL-6 and IL-8 expression in RA synovial tissues [92]. HDAC inhibitors, such as TSA and givinostat, suppress the IL-6 production that is induced by IL-1*β*, TNF*α*, and Toll-like receptor (TLR) ligands [93]. The HDAC inhibitors are suggested to decrease the stability of IL-6 mRNA in RASFs. On the other hand, curcumin, a HAT inhibitor, downregulates IL-6 expression by decreasing the level of H3ac in the IL-6 promoter in RASFs [86].

### 4.2. SLE

The levels of H3K4me3 are altered in key relevant candidate genes, such as PTPN22 and LRP1B, in PBMCs in SLE patients [94]. The CD70 (also known as TNFSF7) gene is highly expressed in SLE T cells and is involved in the synthesis of autoreactive antibodies [95]. Active histone markers, such as H3ac and H3K4me2, in the CD70 promoter were shown to be significantly increased in SLE CD4^+^ T cells and to positively correlate with the disease activity. Both TNF*α* gene expression and histone acetylation at the TNF*α* locus were shown to be enhanced in monocytes of SLE patients [96]. Protein phosphatase 2A (PP2A) is a serine/threonine phosphatase and highly expressed in SLE T cells [97]. Overexpression of PP2A in murine T cells causes glomerulonephritis in an IL-17-dependent manner. IL-17 is produced by T helper 17 cells (Th17) that are implicated in autoimmune diseases. PP2A enhances IL-17 gene expression through H3ac. A genome-wide analysis showed that H4ac is significantly altered in monocytes of SLE patients [98]. Sixty-three percent of genes with increased H4ac are associated with the regulation by interferon regulatory factor 1 (IRF1), suggesting that interferon *α* (IFN*α*) contributes to the pathogenesis of SLE.

Hematopoietic progenitor kinase 1 (HPK1, also called MAP4K1) represses the T cell-mediated immune response [99]. H3K27me3 is enriched in the HPK1 promoter and HPK1 expression is reduced in SLE CD4^+^ T cells. The downregulation of HPK1 results in accelerated T cell proliferation and the production of IFN*γ* and immunoglobulins. The binding of JMJD3 that demethylates H3K27 is decreased, while the binding of EZH2 that methylates H3K27 is not altered in the HPK1 promoter in SLE CD4^+^ T cells. Global hypoacetylation of the histones H3 and H4 has been detected in CD4^+^ T cells of active SLE patients [100]. The level of H3ac is negatively correlated with the disease activity (SLEDAI). Global hypomethylation of H3K9 was observed in CD4^+^ T cells of both active and inactive SLE patients, whereas global H3K4 methylation levels were not altered in SLE CD4^+^ T cells. The gene expression of histone-modifying enzymes was shown to be aberrant in SLE CD4^+^ T cells. SIRT1 gene expression is significantly increased, while CBP, p300, HDAC2, HDAC7, SUV39H2, and EZH2 gene expression is significantly decreased in CD4^+^ T cells of active SLE patients. Regulatory factor X-box 1 (RFX1), which interacts with HDAC1 and SUV39H1, is downregulated in SLE [101, 102]. Therefore, H3ac is increased and H3K9me3 is decreased in the promoters of CD11a and CD70 in SLE CD4^+^ T cells, resulting in CD11a and CD70 overexpression and autoimmune responses. H3K18 deacetylation by HDAC1 results in a silencing of the IL-2 gene in SLE T cells [103]. TSA significantly downregulates CD154 (CD40L) and IL-10 gene expression and upregulates IFN*γ* gene expression in SLE T cells [104].

### 4.3. SSc

The inhibition of H3K27me3 by 3-deazaneplanocin (DZNep) stimulates the release of collagen, induces the profibrotic transcription factor fos-related antigen 2 (FRA-2), and exacerbates the fibrosis induced by transforming growth factor *β* (TGF*β*) in cultured SSc fibroblasts [105]. JMJD3 was reported to be highly expressed and the level of H3K27me3 is decreased in SSc CD4^+^ T cells [106]. As a result, specific genes, such as CD40L, CD70, and CD11a, are activated in SSc, leading to the autoimmune response. Global histone H4 hyperacetylation and histone H3K9 hypomethylation have been reported in SSc B cells [107]. JHDM2A expression is increased, whereas HDAC2, HDAC7, and SUV39H2 expression is decreased in SSc B cells. Global H4ac is negatively correlated with HDAC2 expression. The former was shown to be positively correlated with the disease activity and the latter negatively correlated with skin thickness. Global H3K9 methylation is positively correlated with SUV39H2 expression. Increased collagen synthesis is related to hypoacetylation of the histones H3 and H4 in the collagen suppressor gene FLI1 promoter in SSc fibroblasts [108]. The addition of TSA to cell cultures normalizes collagen expression in SSc fibroblasts. The silencing of HDAC7 using small interfering RNA decreases the production of type I and type III collagen, but not fibronectin, in SSc fibroblasts [109].

### 4.4. PBC

The *β*-Arrestins (*β*arr) are multifunctional signaling molecules that are essential to T cell survival. *β*arr1 expression was shown to be enhanced in PBC T cells [110]. *β*arr1 gene expression is positively correlated with the disease activity (Mayo risk score). The overexpression of *β*arr1 enhances T cell proliferation, increases IFN*γ* production, represses the activities of both NF-*κ*B and activator protein-1 (AP-1), induces H4ac in the CD40L, TNF superfamily member 14 (TNFSF14), IL-17, and IFN*γ* promoters, and suppresses H4ac in the TNF related apoptosis-inducing ligand (TRAIL), Apo2, and HDAC7 promoters, thereby regulating T cell autoreactivity.

### 4.5. T1D

A genome-wide analysis showed differential changes in H3K4me2 and H3K9me2 in monocytes under a high glucose (HG) condition [111]. Furthermore, H3K9me2 is significantly elevated in the phosphatase and tensin homolog deleted from chromosome 10 (PTEN) and IL-1A gene loci in T1D monocytes. The same group showed that the levels of H3K9me2 are altered in several genes, which are associated with the TGF*β*, NF-*κ*B, and IL-6 signaling pathways in T1D lymphocytes by genome-wide analyses [112]. In diabetic patients, inflammation and cardiovascular complications continue even after glycemic control is achieved, suggesting the presence of a “hyperglycemic memory.” IL-6 gene expression is increased and the level of H3K9me3 is decreased in the IL-6 promoter in cardiomyocyte cells in a HG condition [113]. The expression of SUV39H1, an HMT that catalyzes H3K9, is also reduced after HG treatment. The effects of HG on the change in both IL-6 expression and H3K9me3 in the IL-6 gene are irreversible after the removal of HG from the culture. This result is suggested to be associated with hyperglycemic memory in diabetic patients. Hyperglycemia sustained the upregulation of NF-*κ*B (p65) gene expression together with an increase in H3K4me1 but not H3K4me2 or H3K4me3 along with a decrease in H3K9me2 and H3K9me3 in the promoter [114]. Glucose was shown to recruit LSD1, which demethylates H3K4me2 and H3K4me3, to the p65 promoter. Genome-wide analyses revealed that more promoter regions that were enriched in H3K9ac in monocytes were identified in T1D patients than in control subjects [115]. The levels of H3K9ac in monocytes are significantly associated with the levels of glycated hemoglobin (HbA1c), which reflects blood sugar control, in T1D patients. Genes with high H3K9ac levels were shown to be related to the NF-*κ*B signaling pathway. Latent autoimmune diabetes in adults (LADA) is a slow onset form of T1D [116]. Global H3ac but not H4ac is reduced in LADA CD4^+^ T cells. The level of H3ac is correlated with HbA1c in LADA. CBP expression is downregulated, whereas HDAC1 and HDAC7 expression is upregulated in LADA CD4^+^ T cells.

## 5. Conclusion

Increasing evidence has shown that aberrant profiles of histone modifications contribute to the dysregulation of immune response, resulting in the development of a variety of autoimmune diseases. Because there are a number of histone modifications, their functions are complicated and difficult to understand. Further studies are required to break the histone modification code, which is implicated in the pathogenesis of autoimmune diseases. It is hoped that advances in our understanding of the roles of histone modifications in autoimmune diseases will provide a better grasp of the pathogenesis of autoimmune diseases and thus help speed the development of new therapeutic strategies and biomarkers for autoimmune diseases.

## Figures and Tables

**Table 1:** Histone methylation and histone methylation-modifying enzymes.

Histone and resides	States of methylation			HMTs	HDMs
Lysine	me1	me2	me3		

H2BK5	A	—	—	Unknown	Unknown
H3K4	A	A	A	MLL1, MLL2, MLL3, MLL4, ML5, SET1A, SET1B, ASH1L, SET7/9, SMYD3, PRDM9	LSD1, AOF1, FBXL10, JARID1A, JARID1B, JARID1C, JARID1D, JARID2, NO66
H3K9	A	R	R	SUV39H1, SUV39H2, G9A, GLP, SETDB1, SETDB2, PRDM1, PRDM2, PRDM4	LSD1, JMJD1A, JMJD1B, JMJD1C, JMJD2A, JMJD2B, JMJD2C, JMJD2D, PHF8, JHDM1D
H3K27	A	R	R	EZH1, EZH2	UTX, UTY, JMJD3, JHDM1D
H3K36	—	—	A	SETD2, NSD1, NSD2, NSD3, SMYD1, SMYD2, SMYD3, SMYD4, SMYD5	FBXL10, FBXL11, JMJD2A, JMJD2B, JMJD2C, NO66
H3K79	—	—	A or R	DOT1	PHF8
H4K20	A	—	R	PR-Set7, SUV4-20H1, SUV4-20H/2, SET7/9	Unknown

Arginine	me1	me2a	me2s		

H3R2	A	A or R	—	me2a: PRMT6	Unknown
H3R8	—	—	R	me2s: PRMT5	Unknown
H3R17	—	A	—	me2a: CARM1	Unknown
H3R26	—	A	—	me2a: CARM1	Unknown
H4R3	—	A	R	me2a: PRMT1, PRMT6, PRMT8 me2s: PRMT5, PRMT7	Unknown

—: function is unknown, A: an active marker, and R: a repressive marker.

me1: monomethylation, me2: dimethylation, me3: trimethylation, me2s: symmetrical dimethylation, and me2a: asymmetrical demethylation.
